# Salvage Therapy for Relapsed Malignant Pleural Mesothelioma: A Systematic Review and Network Meta-Analysis

**DOI:** 10.3390/cancers14010182

**Published:** 2021-12-30

**Authors:** Yu-Chen Tsai, Hsiao-Ling Chen, Tai-Huang Lee, Hsiu-Mei Chang, Kuan-Li Wu, Cheng-Hao Chuang, Yong-Chieh Chang, Yu-Kang Tu, Jen-Yu Hung, Chih-Jen Yang, Inn-Wen Chong

**Affiliations:** 1 Division of Pulmonary and Critical Care Medicine, Department of Internal Medicine, Kaohsiung Medical University Hospital, Kaohsiung Medical University, Kaohsiung 80708, Taiwan; 1010362@kmuh.org.tw (Y.-C.T.); 1080208@kmuh.org.tw (T.-H.L.); 1070476@kmuh.org.tw (K.-L.W.); 1040239@kmuh.org.tw (C.-H.C.); jyhung@kmu.edu.tw (J.-Y.H.); 2 Department of Internal Medicine, Kaohsiung Municipal Ta-Tung Hospital, Kaohsiung Medical University, Kaohsiung 80145, Taiwan; 3 Department of Pharmacy, Kaohsiung Municipal Ta-Tung Hospital, Kaohsiung Medical University, Kaohsiung 80145, Taiwan; 1058065@kmuh.org.tw (H.-L.C.); 880504@kmhk.org.tw (H.-M.C.); 980770@kmuh.org.tw (Y.-C.C.); 4 Department of Internal Medicine, School of Medicine, College of Medicine, Kaohsiung Medical University, Kaohsiung 80708, Taiwan; 5 Institute of Epidemiology and Preventive Medicine, National Taiwan University, Taipei 10055, Taiwan; yukangtu@ntu.edu.tw; 6 Department of Medical Research, National Taiwan University Hospital, Taipei 10055, Taiwan; 7 Department of Respiratory Therapy, College of Medicine, Kaohsiung Medical University, Kaohsiung 80708, Taiwan; 8 Faculty of Post-Baccalaureate Medicine, College of Medicine, Kaohsiung Medical University, Kaohsiung 80708, Taiwan

**Keywords:** malignant pleural mesothelioma (MPM), network meta-analysis, chemotherapy, NGR-hTNF, vorinostat, anetumab, pembrolizumab, tremelimumab, nivolumab, ipilimumab

## Abstract

**Simple Summary:**

Malignant pleural mesothelioma (MPM) is an aggressive cancer with limited therapeutic options. Pemetrexed plus platinum is a standard first-line therapy, but options for second-line therapy in patients with relapsed mesothelioma remain controversial. Several drugs were recently introduced to treat relapsed MPM. We conducted a comprehensive systematic review and network meta-analysis to evaluate the efficacy of these drugs according to published randomized controlled trials. Nivolumab alone or nivolumab plus ipilimumab provided significantly longer overall survival (OS), and treatment with nivolumab plus ipilimumab was associated with the best OS based on the surface under the cumulative ranking curve (SUCRA). The network meta-analysis revealed that tremelimumab, vorinostat, chemotherapy (CTX), asparagine–glycine–arginine–human tumor necrosis factor plus CTX, nivolumab alone, and nivolumab plus ipilimumab all produced significant progression-free survival (PFS) benefits compared with placebo, with nivolumab plus ipilimumab ranked first for PFS according to SUCRA.

**Abstract:**

Patients with malignant pleural mesothelioma (MPM) have very poor prognoses, and pemetrexed plus platinum is the standard first-line therapy. However, the second-line therapy for relapsed MPM remains controversial. A comprehensive search was performed to identify randomized controlled trials (RCTs) evaluating various second-line regimens in patients with relapsed MPM. Indirect comparisons of overall survival (OS) and progression-free survival (PFS) were performed using network meta-analysis. Surface under the cumulative ranking curve (SUCRA) values were used to rank the included treatments according to each outcome. Nivolumab alone or nivolumab plus ipilimumab provided significantly longer OS than placebo (hazard ratio (HR): 0.72, 95% confidence interval (CI): 0.55–0.94 for nivolumab alone; HR: 0.54, 95% CI: 0.31–0.92 for nivolumab plus ipilimumab). The best SUCRA ranking for OS was identified for nivolumab plus ipilimumab (SUCRA: 90.8%). Tremelimumab, vorinostat, nivolumab alone, chemotherapy (CTX), asparagine–glycine–arginine–human tumor necrosis factor plus CTX, and nivolumab plus ipilimumab all produced noticeable PFS benefits compared with placebo. Nivolumab plus ipilimumab had the best PFS ranking (SUCRA: 92.3%). Second-line treatment with nivolumab plus ipilimumab provided the OS and PFS outcomes for patients with relapsed MPM.

## 1. Introduction

Malignant pleural mesothelioma (MPM) is a rare but aggressive malignancy that originates in pleural mesothelial surfaces. MPM is commonly associated with prior environmental asbestos exposure, with a long latency period, such that decades can pass between asbestos fiber exposure and disease presentation [1]. Asbestos has multiple structural forms, including chrysotile (white asbestos), amosite (brown asbestos), crocidolite (blue asbestos), anthophyllite, tremolite, and actinolite. Chrysotile is the most commonly used form of asbestos in commercial applications. The risk of developing MPM is associated with the type of fiber exposure, in addition to the amount and duration of exposure. [2].

The mechanism underlying carcinogenesis in MPM is multifactorial. Asbestos fibers are inhaled and migrate into the pleura, where they cause irritation and initiate a vicious cycle of tissue injury and repair. Macrophages release oxygen free radicals when they phagocytose asbestos fibers, leading to intracellular DNA damage and abnormal repair. Asbestos fibers also interfere with gene expression in mesothelial cells, altering the chromosome structure. Asbestos-exposed mesothelial cells release inflammatory cytokines, including platelet-derived growth factor, transforming growth factor-β, and vascular endothelial growth factor, establishing a favorable microenvironment for tumor growth, inducing the phosphorylation of various protein kinases and the increased expression of protooncogenes, and promoting abnormal cellular proliferation [3].

Patients with MPM always have very poor prognoses, with a median overall survival (OS) of approximately 12 months from diagnosis [3]. Women have a more favorable outlook than men, but most patients with MPM are men due to the occupational nature of the disease [3]. Four primary histological subtypes have been identified in MPM: epithelioid (60–70%), sarcomatoid (10–15%), the remaining types are biphasic or mixed, and desmoplastic type. The epithelioid subtype is associated with the most favorable prognosis, with a median overall survival of 13.1 months. The sarcomatoid variant is associated with the worst outcomes, with a median survival of just 4 months [3].

In addition, patients with MPM often exhibit resistance to chemotherapy (CTX), and only a few patients are candidates for radical surgery [4]. Surgery is controversial and limited to patients with early-stage disease and good functional performance status. Poor prognosis in patients with MPM has been associated with older age, male sex, high neutrophil-to-lymphocyte ratio, non-epithelial histology, poor Eastern Cooperative Oncology Group (ECOG) performance status, low European Organization for Research and Treatment of Cancer (EORTC) prognostic score, lack of tumor response to previous therapy [5], and a short treatment-free interval between the completion of first-line therapy and the initiation of second-line therapy [6].

In 2003, a phase III trial randomized 448 treatment-naïve participants to receive either pemetrexed and cisplatin or cisplatin alone. Median overall survival for treatment with pemetrexed and cisplatin arm was 12.1 months, compared with 9.3 months for cisplatin alone (*p* = 0.02). Based on this trial, pemetrexed was approved by global authorities for use in combination with cisplatin to treat MPM and remains the standard first-line CTX regimen prescribed for patients with MPM. Another trial compared raltitrexed combined with cisplatin against cisplatin alone in 250 participants. The survival benefit for raltitrexed combined with cisplatin relative to cisplatin alone (11.4 months versus 8.8 months, *p* = 0.048) was similar to that observed for pemetrexed combined with cisplatin but with lower objective response rates, and the trial was underpowered. An evaluation of over 1700 patients who received pemetrexed combined with either cisplatin or carboplatin as part of an expanded access program demonstrated response rates of 26.3% and 21.7%, respectively. Based on these trials, the combination doublet of cisplatin and pemetrexed is currently the standard treatment option for advanced, unresectable MPM [5]. In 2016, an open-label phase 3 RCT trial examined the addition of bevacizumab to the combination of pemetrexed and cisplatin in treatment-naïve malignant mesothelioma, which demonstrated an additional incremental benefit for OS [7].

However, appropriate second-line treatments for relapsed MPM remain controversial. Several cytotoxic CTX agents have been proposed for use as salvage therapy. In addition, immune checkpoint inhibitors (ICIs) have shown promising antitumor activity across various cancer types. Currently, single nivolumab and nivolumab plus ipilimumab have completed clinical trials, and both regimens demonstrated favorable results in relapsed MPM [2]. Several new agents designed to target relapsed mesothelioma have also been developed, included mesothelin-targeted therapies and arginine deprivation for the treatment of arginosuccinate synthetase 1-deficient mesothelioma. Patients with relapsed mesothelioma are always highly resistant to treatment, with fatal outcomes, making the identification of reliably second-line therapies an urgent need. To date, no head-to-head trials have been performed to compare various second-line treatment options. The purpose of this study was to perform a comprehensive systematic review and network meta-analysis (NMA) of currently available clinical trials to compare the benefits of various therapeutic agents in patients with relapsed MPM in terms of PFS and OS.

## 2. Materials and Methods

This systematic review followed the Preferred Reporting Items for Systematic Reviews and Meta-Analyses (PRISMA) 2020 extension statement for network meta-analyses [8]. A protocol was created and registered on The International Prospective Register of Systematic Reviews (PROSPERO) website (Registration No.: CRD42021277641)

### 2.1. Search Strategy and Study Selection

Comprehensive literature searches were performed in the PubMed, Embase, and Clinical-Trials.gov databases through 12 August 2021 without any language limitations. Search terms (MeSH terms, Emtree, and free text words) related to “relapsed mesothelioma”, “second-line”, and “pemetrexed” were used in the search strategy, and detailed information regarding the search strategy can be found in Supplemental Table S1. To obtain the latest information and decrease reporting bias, we also searched abstracts from global oncology congress databases, such as those associated with the American Society of Clinical Oncology (ASCO), European Medical Oncology (ESMO), American Association of Cancer Research (AACR), The European Lung Cancer Virtual Congress (ELCC), and the World Conference on Lung Cancer (WCLC). Additional studies were sought from the reference lists of included studies. The inclusion criteria were as follows: (1) completed phase II or III RCTs involving adults with relapsed mesothelioma; (2) RCTs focused on patients previously treated with pemetrexed-based CTX; and (3) RCTs that performed efficacy comparisons between different second-line therapies or efficacy comparisons between active treatments and placebo.

### 2.2. Data Extraction and Quality Assessment

Two independent reviewers (H.L. Chen and Y.C. Tsai) performed data extraction and quality assessments. Discrepancies were resolved by discussion with a third reviewer (C.J. Yang). Extracted information included RCT name, published year, trial phase, baseline characteristics, treatment arms, subject numbers, OS, and progression-free survival (PFS). Quality assessments were performed using the Risk of Bias (ROB) assessment tool, as recommended by the Cochrane Handbook for Systematic Reviews of Interventions [9].

### 2.3. Data Synthesis and Statistical Analysis

Treatment efficacy was evaluated according to OS and PFS. The adjusted hazard ratio (HR) was considered to be a representation of the effect size for time-dependent indicators, such as OS and PFS. If HR was not provided by the published trials, it was calculated from Kaplan–Meier (K–M) survival curves, based on the algorithm established by Guyot et al. [10]. The algorithm was applied to inverted K–M equations based on digitized curves, time intervals, and reported numbers at risk.

For data synthesis, a network geometry was generated to present the treatment network across all included trials. Each node in the geometry represented a different second-line intervention for relapsed mesothelioma, and the edges between the nodes were regarded as head-to-head comparisons. NMA was then applied. NMA is an extension of pairwise meta-analysis able to provide indirect comparisons between interventions without head-to-head evidence. Additionally, NMA estimates the relative rankings of different interventions to identify which regimens are the best and which are the worst to facilitate clinical decisions [11]. In our studies, NMA was conducted under the frequentist framework using the mvmeta Stata command (version 16, Stata, College Station, TX, USA) [12]. The contrast-based analysis was performed with the restricted maximum likelihood approach to estimate multiple treatment comparisons [13]. The surface under the cumulative ranking curves (SUCRAs) were evaluated to rank all included treatments for each outcome. SUCRA is a numeric presentation ranging from 0% to 100%. A larger SUCRA indicates a higher likelihood that an intervention is associated with the best efficacy or the lowest risk of adverse events. Finally, we checked consistency and transitivity to validate the NMA outcomes. Based on the treatment interaction model, inconsistency was defined as differences in estimates for treatment contrasts between different designs [14]. Transitivity was evaluated by comparing the distribution of common comparators across different comparisons [15].

## 3. Results

### 3.1. Literature Search

**Figure 1:**
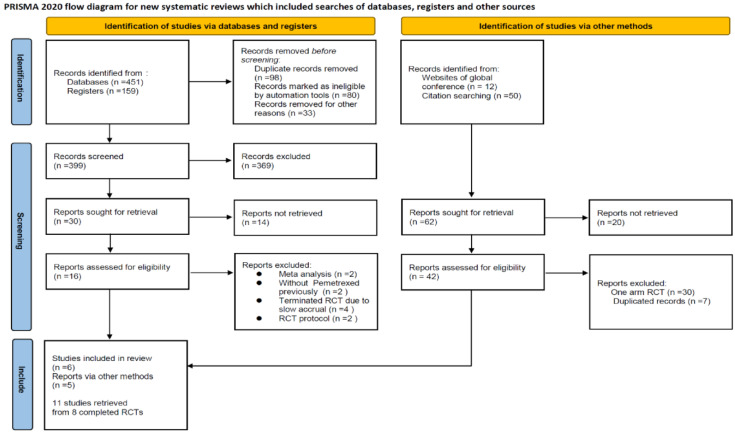
PRISMA flow diagram.

The PRISMA 2020 flow diagram and detailed reasons for study exclusion are presented in Figure 1. A total of 610 studies were imported from databases and registers. After automated screening, title/abstract screening, and full-text review, seven published studies met our inclusion criteria. We also identified 62 studies from the websites associated with global conferences and from the reference lists of the included studies, resulting in the inclusion of five additional studies. At the end of the process, 11 published studies were retrieved from eight completed RCTs, which were retained for qualitative synthesis.

### 3.2. Study Characteristics and Quality Evaluation

The characteristics of included studies are presented in Table 1. All included studies were two-arm phase II or III RCTs conducted for the treatment of relapsed mesothelioma. Involved subjects progressed after previous first-line pemetrexed-based regimens combined with a platinum agent. Among the included studies, four RCTs demonstrated treatment effects for ICIs. Nivolumab (an anti–programmed cell death protein 1 (PD-1) agent) and tremelimumab (an anti–cytotoxic T-lymphocyte–associated protein 4 (CTLA4) agent) were compared against placebo in the CONFIRM [16] and DETERMINE trials, respectively [17]. Pembrolizumab (an anti–PD-1 agent) was compared with CTX (patients with single-drug treatments, such as gemcitabine or vinorelbine) in the PROMISE MESO trial [18]. The IFCT-1501 MAPS2 trial [19] assessed the efficacy for nivolumab alone or in combination with ipilimumab (anti–CTLA4 agent). In addition, three RCTs demonstrated treatment effects associated with targeted therapy. Vorinostat (histone deacetylase (HDAC) inhibitor) was compared with placebo in the VANTAGE-014 trial [20], and anetumab (a mesothelin-targeted antibody) was compared with CTX (vinorelbine) in the MPM trial [21]. The NGR015 trial [22] compared the effects of CTX alone (patients with single-drug CTX, such as doxorubicin, gemcitabine, or vinorelbine) and those of asparagine–glycine–arginine–human tumor necrosis factor (NGR-hTNF, a vascular-targeting drug) plus CTX. Finally, CTX (vinorelbine) was compared with placebo in the VIM trial [23]. The median age across all studies was 62–71 years old, and the percentage of men across the trials ranged from 73% to 85%. In terms of disease sites and histology, more than 95% of subjects were reported as having pleural mesothelioma, and 80–90% subjects were reported as having epithelioid mesothelioma.

The results of the quality assessment are presented in Supplement Figure S1. The protocol for the VIM trial was not provided at the 2021 ASCO annual meeting; therefore, we retrieved study information from an RCT registration website. Studies were identified as having a high risk for performance bias, and an open-label design was conducted in the IFCT-1501 MAPS, PROMISE MESO, MPM, and VIM trials. Quality was unclear in terms of sequence generation, allocation concealment, and selective reporting due to a lack of detailed information.

### 3.3. Efficacy Evaluation from the Network Meta-Analysis

#### 3.3.1. Network Geometry

**Figure 2:**
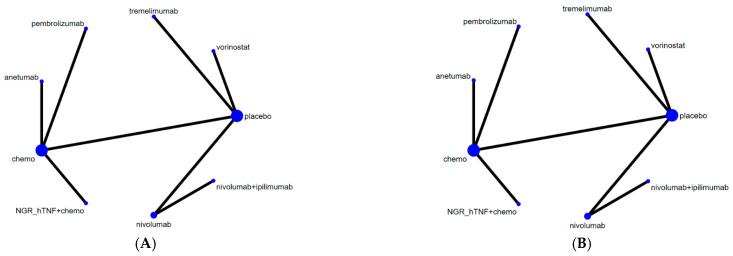
Network constructions for comparisons of overall survival (OS) and progression-free survival (PFS). (**A**) Network constructions for the comparison of OS (based on hazard ratios (HRs)). (**B**) Network constructions for the comparison in PFS (HR).

Because different CTX drugs were tested in the CTX arms of the PROMISE MESO and NGR015 trials, we first regarded all CTX drugs as the CTX group and determined the network geometries for OS (Figure 2A) and PFS (Figure 2B). Placebo, CTX, NGR-hTNF plus CTX, vorinostat, anetumab, pembrolizumab, tremelimumab, nivolumab plus ipilimumab, and nivolumab alone are included in Figure 2A,B.

After excluding the PROMISE MESO and NGR015 trials, we performed a sensitivity analysis for the remaining individual chemotherapy agents. Supplemental Figure S2 presents the network geometries obtained for the OS and PFS associated with vinorelbine (a vinca alkaloid chemotherapy), placebo, vorinostat, anetumab, tremelimumab, nivolumab plus ipilimumab, and nivolumab alone.

#### 3.3.2. Overall Survival

**Figure 3:**
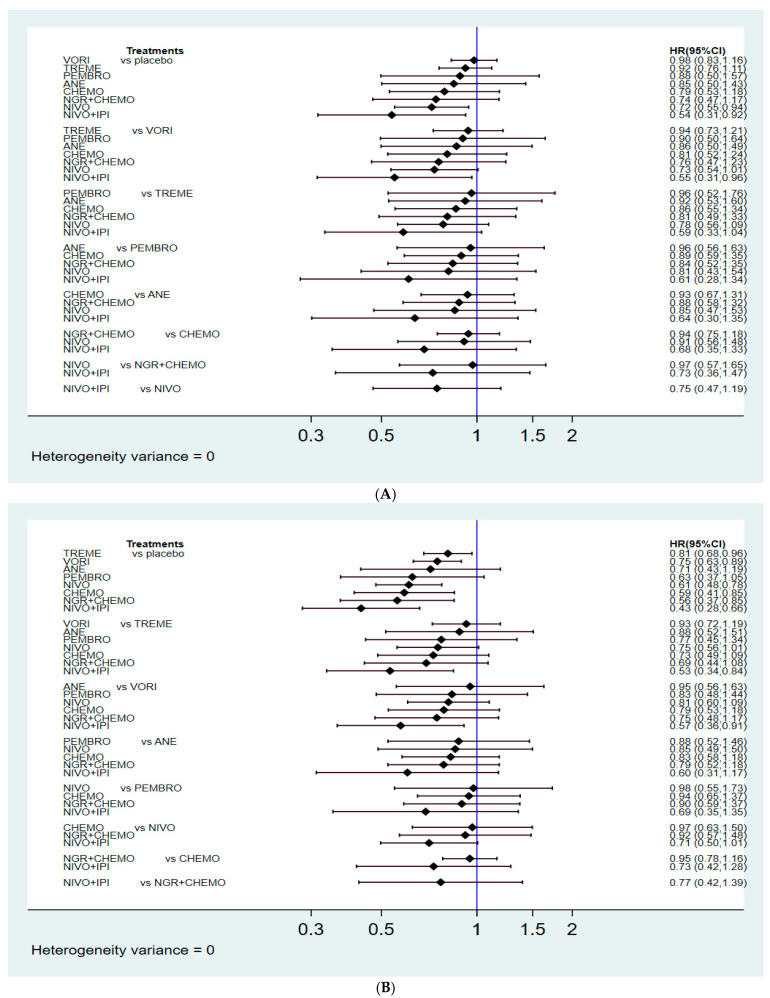
Summary of effect sizes based on pairwise comparisons. (**A**) Hazard ratios for overall survival (OS); (**B**) Hazard ratio for progression-free survival (PFS).

The OS results are presented in Figure 3A. Patients who received nivolumab alone or nivolumab plus ipilimumab were associated with significantly longer OS than those who received placebo (HR: 0.72, 95% confidence interval (CI): 0.55–0.94 for nivolumab alone; HR: 0.54, 95% CI: 0.31–0.92 for nivolumab plus ipilimumab) among all enrolled regimens. Although no superior effects were indicated, other active treatments, including vorinostat, tremelimumab, pembrolizumab, anetumab, CTX, and NGR-hTNF plus CTX, presented with relatively lower HRs compared with placebo (HR: 0.98, 95% CI: 0.83–1.16 for vorinostat; HR: 0.92, 95% CI: 0.76–1.11 for tremelimumab; HR: 0.88, 95% CI: 0.50–1.57 for pembrolizumab; HR: 0.85, 95% CI: 0.50–1.43 for anetumab; HR: 0.79, 95% CI: 0.53–1.18 for CTX; HR: 0.74, 95% CI: 0.47–1.17 for NGR-hTNF plus CTX).

In addition, we found no significant differences between CTX alone and other second-line treatment agents. However, the survival analysis associated with NGR-hTNF plus CTX, nivolumab alone, and nivolumab plus ipilimumab produced relatively lower HR values than CTX alone (HR: 0.94, 95% CI: 0.75–1.18 for NGR-hTNF plus CTX; HR: 0.91, 95% CI: 0.56–1.48 for nivolumab alone; HR: 0.68, 95% CI: 0.35–1.33 for nivolumab plus ipilimumab).

No significant differences were presented among targeted therapies. The survival benefits associated with anetumab and NGR-hTNF plus CTX were both non-inferior to those for vorinostat (HR: 0.86, 95% CI: 0.50–1.49 for anetumab; HR: 0.76, 95% CI: 0.47–1.23 for NGR-hTNF plus CTX). Additionally, similar effects were observed comparing between anetumab and NGR-hTNF plus CTX (HR: 0.88, 95% CI: 0.58–1.32). Although no superior effects were indicated between ICIs, patients who received nivolumab showed a trend toward improvement in terms of OS compared with those who received pembrolizumab and tremelimumab (HR: 0.81, 95% CI: 0.43–1.54 for pembrolizumab; HR: 0.78, 95% CI: 0.56–1.09 for tremelimumab).

Notably, nivolumab plus ipilimumab presented a significantly longer OS than vorinostat (HR: 0.55, 95% CI: 0.31–0.96) and a trend toward longer OS compared with other ICIs (HR: 0.59, 95% CI: 0.33–1.04 for tremelimumab; HR: 0.61, 95% CI: 0.28–1.34 for pembrolizumab; HR: 0.75, 95% CI: 0.47–1.19 for nivolumab). Nivolumab plus ipilimumab trended toward being the best regimen for OS among all other second-line regimens for relapsed malignant mesothelioma in the NMA.

**Figure 4:**
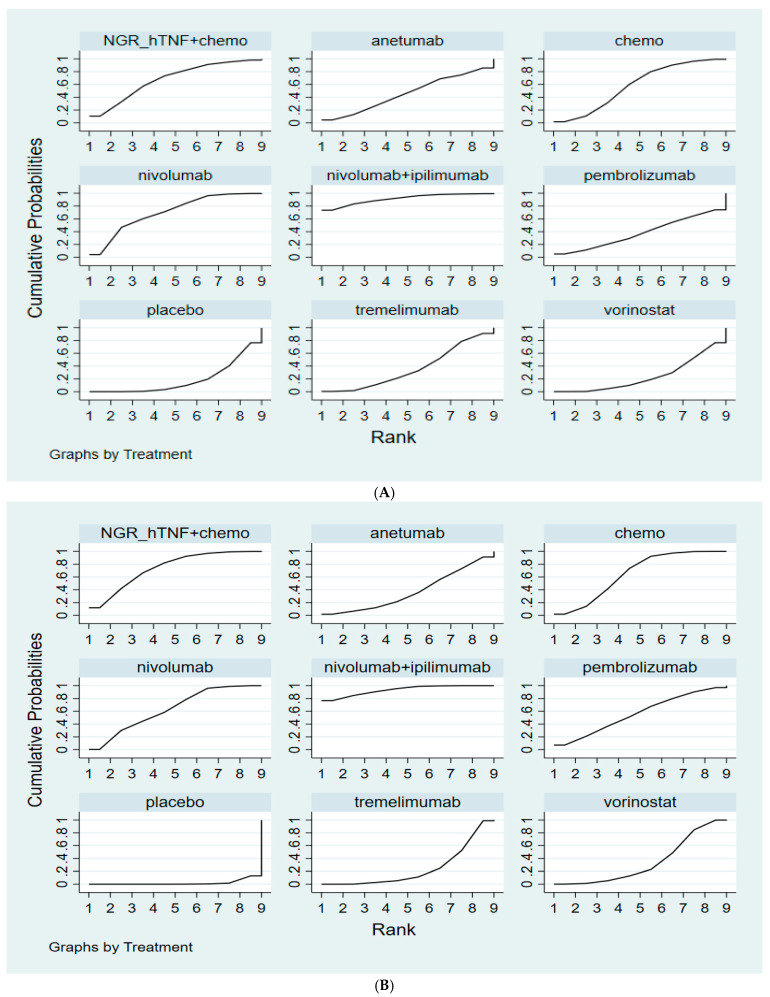
Cumulative ranking probability for different interventions. (**A**) Hazard ratio for overall survival (OS). (**B**) Hazard ratio for progression-free survival (PFS).

The SUCRA rankings are shown in Figure 4A, indicating that nivolumab plus ipilimumab was associated with the best ranking for OS (SUCRA: 90.8%), followed by nivolumab (SUCRA: 69.8%), NGR-hTNF plus CTX (SUCRA: 66.4%), CTX (SUCRA: 56.3%), anetumab (SUCRA: 45.3%), pembrolizumab (SUCRA: 42.2%), tremelimumab (SUCRA: 36.4%), vorinostat (SUCRA: 23.7%), and placebo (SUCRA: 19.1%).

#### 3.3.3. Progression-Free Survival

The PFS results are presented in Figure 3B. Tremelimumab, vorinostat, nivolumab alone, CTX, NGR-hTNF plus CTX, and nivolumab plus ipilimumab produced noticeable benefits over placebo (HR: 0.81, 95% CI: 0.68–0.96 for tremelimumab; HR: 0.75, 95% CI: 0.63–0,89 for vorinostat; HR: 0.61, 95% CI: 0.48–0.78 for nivolumab alone; HR: 0.59, 95% CI: 0.41–0.85 for CTX; HR: 0.56, 95% CI: 0.37–0.85 for NGR-hTNF plus CTX; and HR: 0.43, 95% CI: 0.28–0.66 for nivolumab plus ipilimumab).

Although no superior effects were indicated between CTX and other second-line active treatments, combination therapy presented lower HRs compared with CTX alone (HR: 0.95, 95% CI: 0.78–1.16 for NGR-hTNF plus CTX; HR: 0.73, 95% CI: 0.42–1.28 for nivolumab plus ipilimumab). By contrast, CTX alone had better performance than monotherapy with ICIs or targeted agents (HR: 0.73, 95% CI: 0.49–1.09 for tremelimumab; HR: 0.79, 95% CI: 0.53–1.18 for vorinostat; HR: 0.83, 95% CI: 0.58–1.18 for anetumab; HR: 0.94, 95% CI: 0.65–1.37 for pembrolizumab; and HR: 0.97, 95% CI: 0.63–1.50 for nivolumab alone).

Apart from CTX, comparable PFS outcomes were reported among patients treated with monotherapy using ICIs or targeted agents. However, patients who received combination nivolumab plus ipilimumab were associated with longer PFS than those who received tremelimumab or vorinostat (HR: 0.53, 95% CI: 0.34–0.84 for tremelimumab; HR: 0.57, 95% CI: 0.36–0.91 for vorinostat). Combination therapy using nivolumab plus ipilimumab showed better PFS than nivolumab alone, although the difference was not significant (HR: 0.71, 95% CI: 0.50–1.01). Combination therapy using nivolumab plus ipilimumab appeared to be the most reliable regimen for PFS in the NMA.

As shown in Figure 4B, nivolumab plus ipilimumab had the best SUCRA profile for PFS (SUCRA: 92.3%), followed by NGR-hTNF plus CTX (SUCRA: 73.1%), CTX (SUCRA: 65.0%), nivolumab alone (SUCRA: 62.5%), pembrolizumab (SUCRA: 57.8%), anetumab (SUCRA: 38.7%), vorinostat (SUCRA: 34.5%), tremelimumab (SUCRA: 24.2%), and placebo (SUCRA: 2.0%).

#### 3.3.4. Sensitivity Analysis

Only vinorelbine was regarded as CTX exposure in the sensitivity analysis, and the OS performance of vinorelbine is shown in Supplemental Figure S3. Although no significant differences were reported in OS among all study groups according to the network geometry, nivolumab alone and nivolumab plus ipilimumab produced better OS outcomes than vinorelbine (HR: 0.91, 95% CI: 0.56–1.48 for nivolumab alone; HR: 0.68, 95% CI: 0.35–1.33 for nivolumab plus ipilimumab). By contrast, vinorelbine produced a better OS profile than placebo, vorinostat, tremelimumab, and anetumab (HR: 0.79, 95% CI: 0.53–1.18 for placebo; HR: 0.81, 95% CI: 0.52–1.24 for vorinostat; HR: 0.86, 95% CI: 0.55–1.34 for tremelimumab; HR: 0.93, 95% CI: 0.97–1.31 for anetumab). Similar SUCRA rankings were reported in the sensitivity analysis for OS (Supplemental Figure S4). Nivolumab plus ipilimumab was regarded as having better OS, with the highest SUCRA value (92.7%), followed by nivolumab alone (SUCRA: 71.6%), vinorelbine (SUCRA: 61.2%), anetumab (SUCRA: 49.4%), tremelimumab (SUCRA: 36.8%), vorinostat (SUCRA: 22.5%), and placebo (SUCRA: 15.8%).

In terms of PFS (Supplemental Figures S3B and S4B), vinorelbine produced a noticeable benefit over placebo (HR: 0.59, 95% CI: 0.41–0.85); however, vinorelbine was comparable to tremelimumab, vorinostat, anetumab, nivolumab alone, and nivolumab plus ipilimumab (HR: 0.73, 95% CI: 0.49–1.09 for tremelimumab; HR: 0.79, 95% CI: 0.53–1.18 for vorinostat; HR: 0.83, 95% CI: 0.58–1.18 for anetumab; HR: 0.97, 95% CI: 0.63–1.50 for nivolumab; HR: 0.73, 95% CI: 0.42–1.28 for nivolumab plus ipilimumab compared to vinorelbine). Additionally, nivolumab plus ipilimumab was regarded as having better PFS, with the highest SUCRA value (95.6%), followed by vinorelbine (SUCRA: 73.1%), nivolumab (SUCRA: 66.8%), anetumab (SUCRA: 46.7%), vorinostat (SUCRA: 38.3%), tremelimumab (SUCRA: 27.6%), and placebo (SUCRA: 1.8%).

#### 3.3.5. Consistency and Transitivity

Based on Figure 2 and Figure S2, the network plots showed that each treatment contrast was formed by a two-arm trial; therefore, for all treatment contrasts in our study, direct and indirect evidence came from the same trial. Accordingly, the evidence was always consistent by definition.

According to Table 1, a balanced distribution was presented at baseline among patients who received CTX in the PROMISE MESO, NGR015, MPM, and VIM trials. A balanced distribution was also presented among patients who received placebo in the DETERMINE [17], CONFIRM, VANTAGE-014, and VIM trials. Consequently, CTX and placebo were allowed to serve as common comparators for valid network analysis.

## 4. Discussion

In this study, we conducted a comprehensive systematic review and NMA of second-line therapy for patients with relapsed MPM. To date, for those patients with MPM who relapsed after treatment with first-line pemetrexed plus platinum therapy, the optimal relapse treatment strategy has remained controversial and in dispute. Several treatment regimens have been proposed by clinical trials, with common regimens including CTX alone, immunotherapy, and the other anticancer agents that act through different mechanisms.

By instigating chronic inflammation and localized tumor immunosuppression, the immune system plays a crucial role in the pathogenesis of MPM, and improved outcomes have correlated with increased levels of intra-tumor infiltration by CD8^+^ cytotoxic T cells [19]. ICI therapy is also thought to have a biologically attractive potential benefit for malignant mesothelioma based on the pathogenic inflammatory microenvironment and programmed cell death ligand 1 (PD-L1) expression identified in 14–59% of tumors. High PD-L1 expression in tumors has also been associated with poor prognosis in mesothelioma [19]. Among immunotherapy regimens, the combination of nivolumab plus ipilimumab appeared to demonstrate a better outcome than single nivolumab and or single pembrolizumab for both PFS and OS. Both single nivolumab and the combination of nivolumab plus ipilimumab were found to have better OS than NGR-hTNF plus CTX, but only the combination of nivolumab plus ipilimumab also demonstrated better PFS than NGR-hTNF plus CTX. Malignant mesothelioma is associated with lymphocyte infiltration [24], including regulatory T cells, which produce inhibitory cytokines that induce a highly immunosuppressive environment within the tumor [24]. Targeting PD-1 using ICIs, such as the humanized IgG4 therapeutic antibody nivolumab, has been demonstrated to serve as a useful monotherapy in the relapsed treatment setting [19,25,26]. Nivolumab demonstrated significantly longer PFS compared with placebo in relapsed malignant mesothelioma (median PFS: 3.0 vs. 1.8 months, HR: 0.61, *p* < 0.001) in the CONFIRM trial. However, no improvement was observed for independently reviewed PFS following pembrolizumab treatment compared with CTX with gemcitabine or vinorelbine (median PFS 2.5 vs. 3.4 months, HR: 1.06, 95% CI: 0.73–1.53, *p* = 0.76), and no OS improvement for pembrolizumab over CTX (HR: 1.04, 95% CI: 0.66–1.67, *p* = 0.85) in the PROMISE MESO trial [18].

CTLA4 is a co-inhibitory receptor expressed on T cells that blocks interactions with antigen-presenting cells and reduces the amplitude of CD28-mediated T-cell activation by competitively binding with CD80 (B7-1) and CD86 (B7-2) ligands [27]. CTLA4 blockade enhances T-cell activation and might be associated with antitumor immune responses. The CTLA4 inhibitor ipilimumab is associated with durable survival benefits in patients with metastatic melanoma [28]. Improved OS, PFS, and objective response rate (ORR) were demonstrated in head-to-head comparisons between double immunotherapy featuring ipilimumab combined with nivolumab and nivolumab monotherapy (median OS: 15.9 vs. 11.9 months, HR: 0.75, 95% CI: 0.47–1.19; median PFSL 5.6 vs. 4.0 months, HR: 0.71, 95% CI: 0.50–1.01, ORR: 25.81% vs. 17.46%) in the IFCT-1501 MAPS2 trial [19]. Tremelimumab is a selective human immunoglobulin G2 monoclonal antibody against CTLA4, which promotes T-cell activity but does not deplete regulatory T cells [29]. Tremelimumab did not significantly prolong OS compared with placebo in patients with previously treated malignant mesothelioma (median OS: 7.7 vs. 7.3 months, HR: 0.92, 95% CI: 0.76–1.12, *p* = 0.41) in the DETERMINE trial [17].

In addition to cytotoxic chemotherapy and ICI agents, several new agents were recently designed to treat MPM. Anetumab ravtansine comprises a human anti-mesothelin IgG1 antibody conjugated via a disulfide-containing linker to the maytansinoid tubulin inhibitor DM4, which disrupts microtubule function and inhibits mitosis [30]. Mesothelin, a glycosylphosphatidylinositol (GPI)-anchored glycoprotein, is a tumor differentiation antigen frequently expressed at high levels in tumors, such as mesothelioma, ovarian, pancreatic, and lung adenocarcinomas, and showing restricted expression in nonmalignant tissues [31]. Thus, mesothelin is an attractive target for anticancer therapy that can be targeted using antibody–drug conjugates (ADCs) that combine the specificity of an antibody with the potency of a toxophore. Anetumab ravtansine has promising antitumor activity in mesothelin-expressing solid tumors, such as mesothelioma, ovarian cancer, breast cancer, non-small-cell lung cancer, and pancreatic cancer [31].

Vorinostat (suberoylanilide hydroxamic acid) was the first HDAC inhibitor approved for the treatment of cancer. The modification of histones through acetylation is controlled by the balance between HDACs and histone acetyltransferases [32]. Histone acetylation alters gene expression and protein activity, and aberrant gene expression is caused by increased HDAC activity and histone hypoacetylation in cancer [33]. HDAC inhibitors appear to be promising anticancer drugs, particularly when combined with other anticancer therapies. In 2006, vorinostat was approved for the treatment of cutaneous manifestations in patients with advanced primary cutaneous T-cell lymphoma [34].

NGR-hTNF conjugates human TNFα with the CNGRCG peptide, which targets a CD13 isoform specifically expressed by angiogenic vessels [35]. NGR-hTNF modifies the tumor microenvironment through the NGR motif. Through the improved permeabilization of newly formed tumor vasculature, NGR-hTNF increases the penetration of intratumoral CTX and leukocyte trafficking [22,36]. NGR-hTNF induces apoptosis in both tumor and endothelial cells in vivo, reducing tumor growth [35]. NGR-hTNF has been utilized as an anticancer drug, either alone or in combination with CTX. In the NGR015 trial, the OS did not differ (median OS: 8.5 months; 95% CI: 7.2–9.9 months in the NGR-hTNF group vs. 8.0 months; 95% CI: 6.6–8.9 months in the placebo group; HR: 0.94, 95% CI: 0.75–1.18; *p* = 0.58) [22].

Using vorinostat [20] or the CTLA4 inhibitor tremelimumab [17] in pemetrexed-pretreated patients did not show any survival benefits compared with placebo. Although single cytotoxic CTX (mostly gemcitabine or vinorelbine) is routinely utilized as salvage therapy in patients with relapsed malignant mesothelioma, based on data from single-arm phase 2 studies [23], no available evidence suggests that these drugs provide survival benefits.

Compared with CTX (regimens including gemcitabine, vinorelbine, and doxorubicin), only NGR-hTNF plus CTX and the combination of nivolumab plus ipilimumab resulted in better PFS. Only single nivolumab, the combination of nivolumab plus ipilimumab, and NGR-hTNF plus CTX resulted in better OS than CTX.

According to the current NMA, all regimens resulted in significantly longer PFS than placebo, but none of the examined treatments were remarkable in the setting of anetumab and pembrolizumab. Only ICIs, both single nivolumab and the combination of nivolumab plus ipilimumab resulted in significantly longer OS. The result of current NMA needs further comprehensive head-to -head trials to confirm. 

There were several limitations in our study. First, we had tried our best to find the randomized trials for relapsed MPM in the literature, but only a few articles or studies fit the inclusion criteria. This meta-analysis is relatively small and was only useful for analysis of the available information. Second, the heterogenicity of studies cannot be avoided completely in these enrolled data and study designs. Therefore, we tried to connect these available data by network meta-analysis, and compare the effectiveness by SUCRA value. The regimens of CTX included gemcitabine, vinorelbine, and doxorubicin among the included studies, but we put all the chemotherapy regiments together for further analysis. Third, due to comparable effects were presented in MPM second line therapy, safety issue was another important concern for decision making. However, we cannot pool the safety data in our NMA because wide variety of adverse effects were reported by different severity criteria in different kinds of anticancer drugs. Fourth, all relevant measures for cost-effectiveness in the oncology management would be taken into account by comprehensive evaluation rather than only cost comparisons for different agents. In this analysis, we did not include cost as a parameter, but we believed further cost effectiveness analysis is urgent for decision making, insurance issue and clinical setting.

## 5. Conclusions

Relapsed MPM patients who received nivolumab alone or nivolumab plus ipilimumab both demonstrated significantly longer OS compared with patients who received placebo. Nivolumab plus ipilimumab was associated with the best OS ranking by SUCRA (SUCRA: 90.8%), followed by nivolumab (SUCRA: 69.8%). Tremelimumab, vorinostat, nivolumab alone, CTX, NGR-hTNF plus CTX, and nivolumab plus ipilimumab all produced significant PFS benefits compared with patients who received placebo, and nivolumab plus ipilimumab had the best ranking for PFS in the NMA.

Among all regimens, NGR-hTNF plus CTX, Nivolumab, and combination nivolumab plus ipilimumab all demonstrated trend improvement in OS relative to CTX. Furthermore, NGR-hTNF plus CTX and combination nivolumab plus ipilimumab both showed superior efficacy in PFS relative to CTX, but it was not significant.

## Figures and Tables

**Table 1:** Characteristics of the included studies.

Author	Fennell et al. [16]		Miao et al. [17]		Papot et al. [18]		Scherpereel et al. [19]	
Year	2020		2017		2020		2019	
RCT name	CONFIRM		DETERMINE		PROMISE MESO		IFCT-1501 MAPS2	
NCT number	NCT03063450		NCT01843374		NCT02991482		NCT02716272	
Phase	Phase 3		Phase 2		Phase 3		Phase 2	
Comparison	Nivolumab	Placebo	Tremelimumab	Placebo	Pembrolizumab	Single CTX ^1^	Nivolumab + ipilimumab	Nivolumab
Sample size	221	111	382	189	73	71	62	63
Baseline characteristics								
Age (mean or median)	70	71	66	67	69	71	62	63
Sex (%)								
Male	76.00%	77.00%	74%	80%	79.40%	84.50%	85%	75%
Female	24.00%	23.00%	26%	20%	20.60%	15.50%	15%	25%
Disease site (%)								
Pleural	NA,	NA,	95%	96%	100.00%	100.00%	NA,	NA,
Peritoneal	NA,	NA,	5%	4%			NA,	NA,
Histology (%)								
Epithelioid	88.00%	88.00%	83%	83%	90.40%	87.30%	85%	83%
Non-epithelioid	12.00%	12.00%	17%	17%	9.60%	12.70%	15%	17%
ECOG status (%)								
0	20%	20%	28%	30%	28.80%	19.70%	40%	30%
>0	80%	80%	70%	70%	71.20%	80.30%	60%	67%
**Author**	**Krug et al.** [20]		**Hassan et al.** [21]		**Gregorc et al.** [22]		**Fennel et al.** [23]	
Year	2015		2017		2018		2021	
RCT name	VANTAGE-014		MPM		NGR015		VIM	
NCT number	NCT00128102		NCT02610140		NCT01098266		NCT02139904	
Phase	Phase 3		Phase 2		Phase 3		Phase 2	
Comparison	Vorinostat	Placebo	Anetumab	Vinorelbine	NGR-hTNF + single CTX ^2^	Single CTX ^2^	Vinorelbine	Placebo
Sample size	329	332	166	82	200	200	98	56
Baseline characteristics								
Age (mean or median)	64	65	66.1	65.6	65	67	70.5	70.7
Sex (%)								
Male	86.00%	81.00%	73.50%	75.60%	78.00%	73.00%	81.60%	80.40%
Female	14.00%	19.00%	26.50%	24.40%	22.00%	28.00%	18.40%	19.60%
Disease site (%)								
Pleural	100.00%	100.00%	NA,	NA,	NA,	NA,	100.00%	100.00%
Peritoneal			NA,	NA,	NA,	NA,		
Histology (%)								
Epithelioid	83.00%	81.00%	NA,	NA,	85.00%	82.00%	82.70%	85.70%
Non-epithelioid	17.00%	19.00%	NA,	NA,	15.00%	18.00%	17.30%	14.30%
ECOG performance status (%)								
0	NA,	NA,	NA,	NA,	NA,	NA,	26.50%	21.40%
>0	NA,	NA,	NA,	NA,	NA,	NA,	73.50%	78.60%

Abbreviations: RCT, randomized controlled trial; NCT, National Clinical Trial; CTX, chemotherapy; ECOG, Eastern Cooperative Oncology Group. ^1^ CTX: chemotherapy, single-agent CTX included doxorubicin, gemcitabine, or vinorelbine. NA, non-available, NA, non-available. ^2^ Single CTX: only one chemotherapy agent was used.

## Supplementary Materials

The following are available online at www.mdpi.com/article/10.3390/cancers14010182/s1, Figure S1: Quality assessment by the Risk of Bias (ROB) tool, Figure S2: Network constructions for comparisons of overall survival (OS) and progression-free survival (PFS). (A) Network constructions for comparisons of OS (hazard ratio [HR]). (B) Network constructions for comparisons of PFS (HR), Figure S3: Summary of effect sizes for pairwise comparisons. (A) Hazard ratio for overall survival (OS). (b) Hazard ratio for progression-free survival (PFS), Figure S4: Cumulative ranking scores for the different interventions. (A) Hazard ratio for overall survival (OS). (B) Hazard ratio for progression-free survival (PFS), Table S1: Search strategy.
