# Supplementary material for: Salvage Therapy for Relapsed Malignant Pleural Mesothelioma: A Systematic Review and Network Meta-Analysis
Source: Cancers (Basel). 2021 Dec 30;14(1):182. doi: 10.3390/cancers14010182 (PMC8750103; doi:10.3390/cancers14010182)
Supplement: Supplementary file 1 [file cancers-14-00182-s001.zip › cancers-1503758-supplementary.pdf]

Supplementary materials: Salvage Therapy for Relapsed Malignant Pleural Mesothelioma: A Systematic Review and Network Meta-Analysis

Yu-Chen Tsai, Hsiao-Ling Chen, Tai-Huang Lee, Hsiu-Mei Chang, Kuan-Li Wu, Cheng-Hao Chuang, Yong-Chieh Chang, Yu-Kang Tu, Jen-Yu Hung, Chih-Jen Yang and Inn-Wen Chong

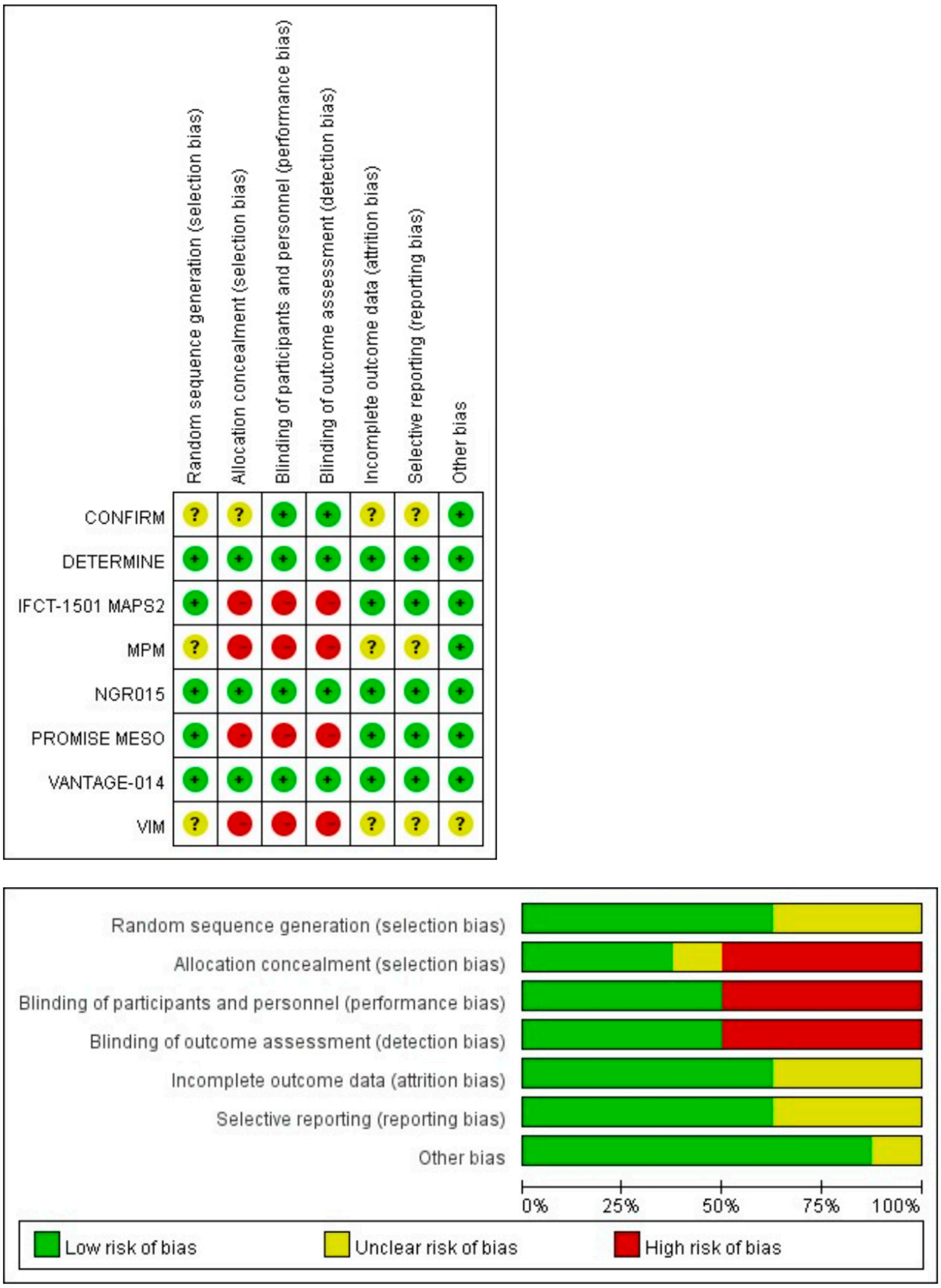

Figure S1. Quality assessment by the Risk of Bias (ROB) tool.

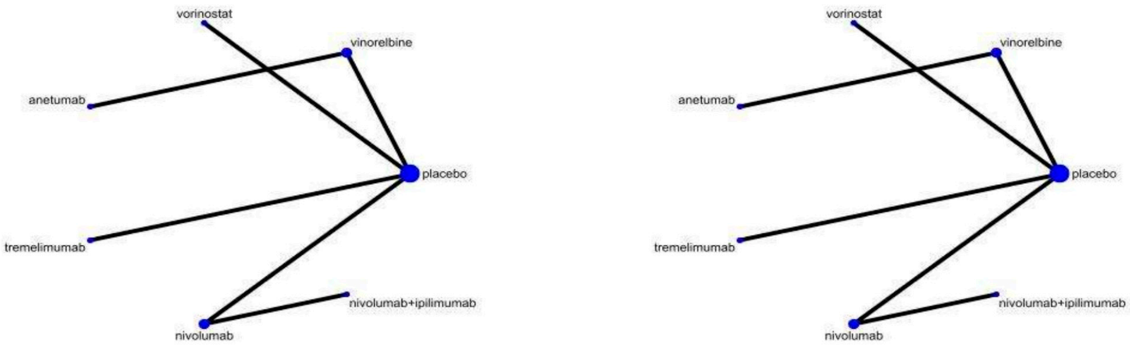

Figure S2. Network constructions for comparisons of overall survival (OS) and progression-free survival (PFS). (**A**) Network constructions for comparisons of OS (hazard ratio [HR]). (**B**) Network constructions for comparisons of PFS (HR).

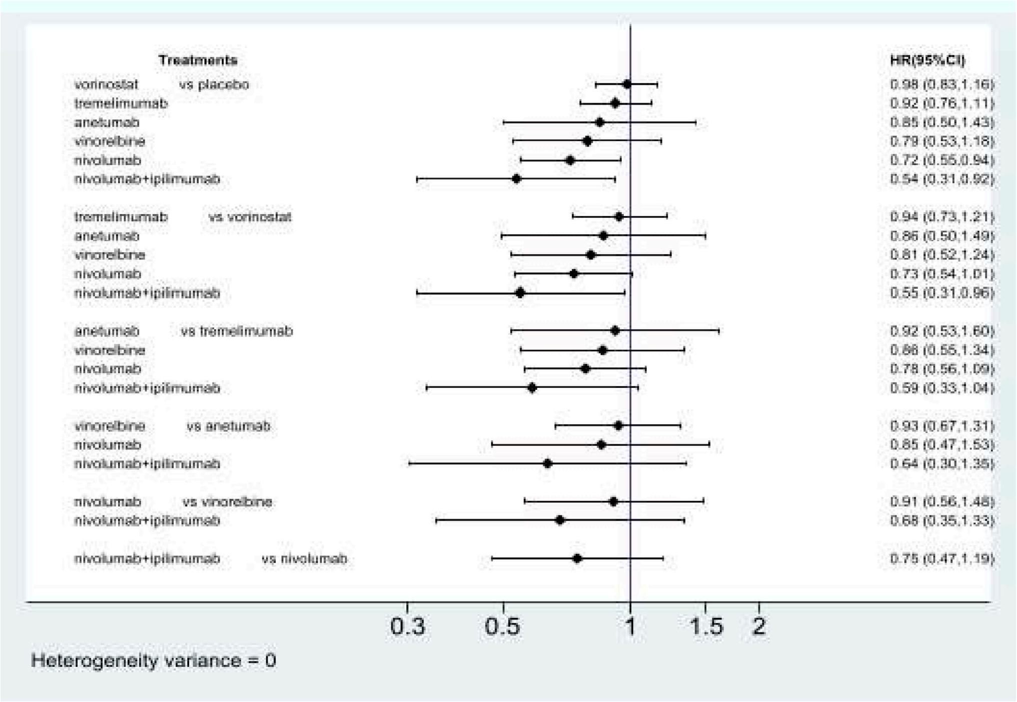

(**A**)

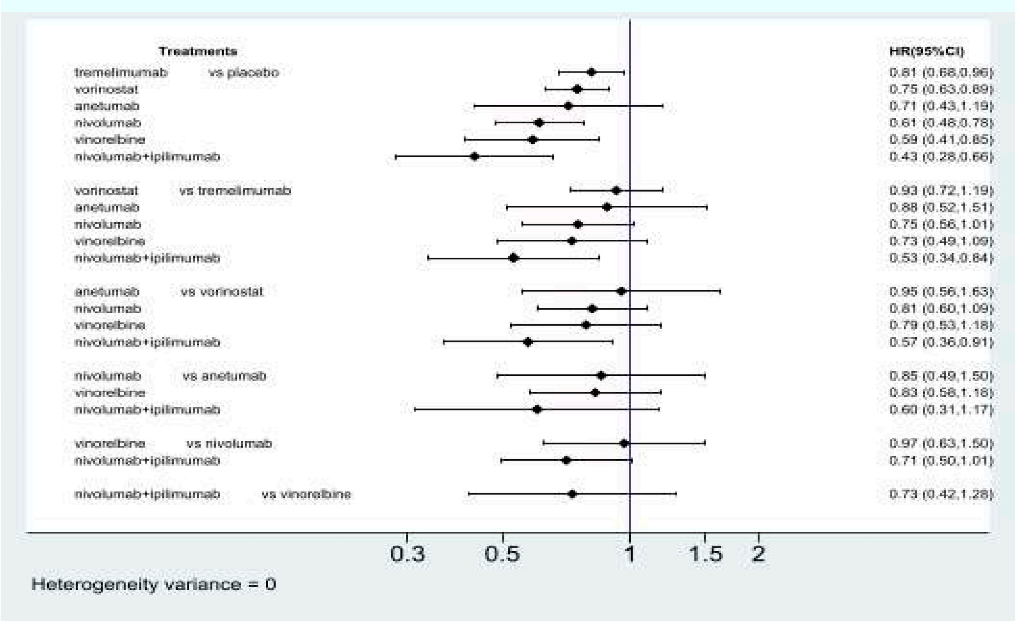

(**B**)

Figure S3. Summary of effect sizes for pairwise comparisons. (**A**) Hazard ratio for overall survival (OS). (**B**) Hazard ratio for progression-free survival (PFS).

 https://doi.org/10.3390/cancers14010182 www.mdpi.com/journal/cancers

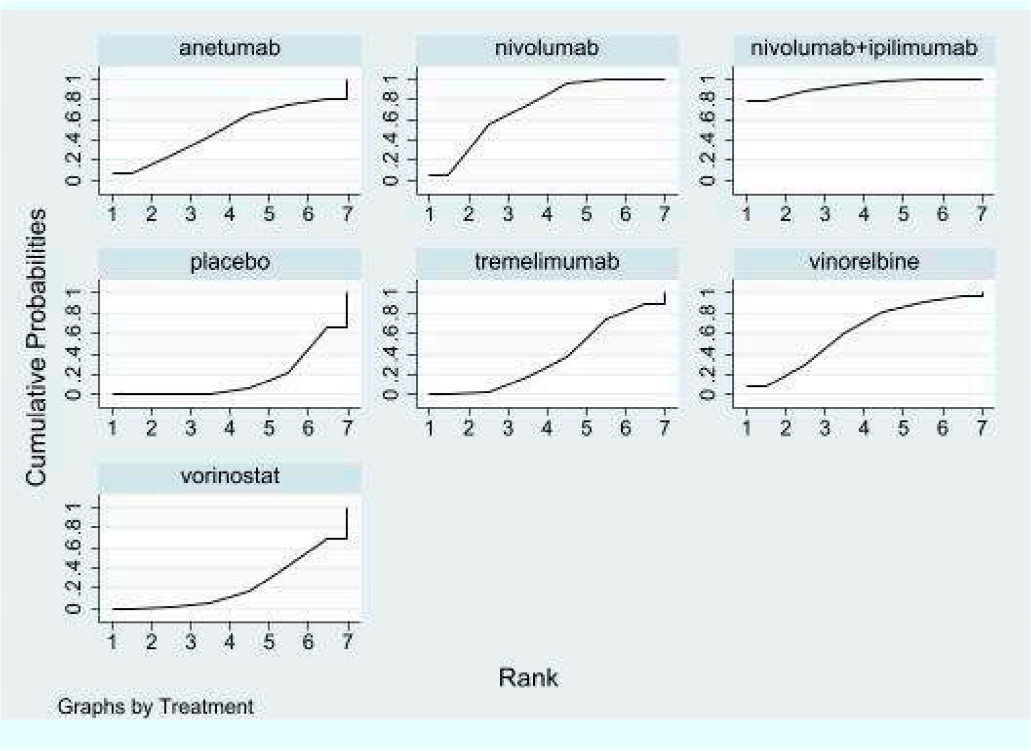

(**A**)

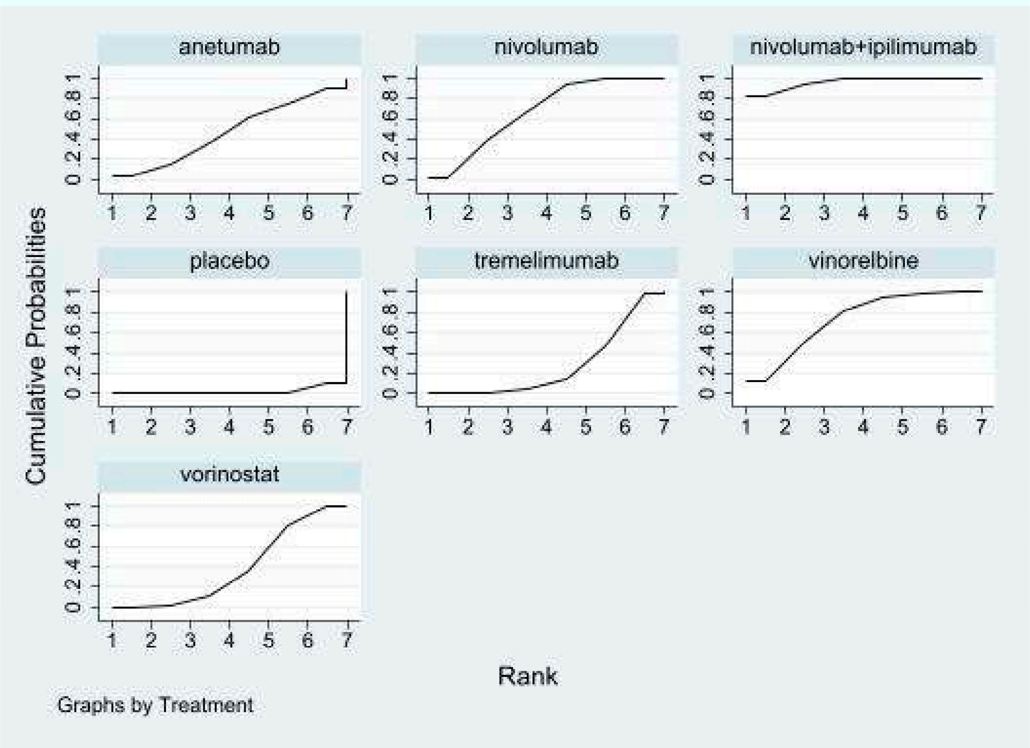

(**B**)

Figure S4. Cumulative ranking scores for the different interventions. (**A**) Hazard ratio for overall survival (OS). (**B**) Hazard ratio for progression-free survival (PFS).

Table S1. Search strategy. Search strategy in Embase ®

- The Embase database: Contains biomedical literature from 1974 to present.

- The MEDLINE & PubMed database: Covers journals from 1966 to present.
- Embase Classic: The Embase back file covers almost 2 million biomedical and pharmacological citations drawn from over 3,000 international titles between 1947 and 1973.

Search	Query	Results
#1	'mesothelioma'/exp OR 'celothelioma' OR 'cystic mesothelioma' OR 'malignant mesothelioma' OR 'mesothelial neoplasms' OR 'mesothelioma' OR 'mesothelioma, cystic' OR 'mesothelioma, malignant' OR 'mesotheliomatosis' OR 'neoplasms, mesothelial' OR 'submesothalial cancer'	30,966
#2	mesothelioma:ab,ti OR ((malignant NEAR/3 mesothelioma*):ab,ti) OR ((neoplasm* NEAR/3 mesothelia*):ab,ti)	23,406
#3	#1 OR #2	31,043
#4	relapse*: ab,ti OR refractory:ab,ti OR progress*:ab,ti OR ((second* NEAR/2 line):ab,ti) OR ((third* NEAR/2 line):ab,ti)	1,990,458
#5	'pemetrexed'/exp OR 'alimta' OR 'pemetrexed' OR 'pemetrexed disodium' OR 'pemetrexed disodium hemipentahydrate' OR 'pemetrexed ditromethamine' OR 'pemetrexed sodium' OR 'pemetrexed tromethamine'	16,207
#6	#3 AND #4 AND #5	1057
#7	'clinical trial'/de OR 'randomized controlled trial'/de OR 'randomization'/de OR 'single blind procedure'/de OR 'double blind procedure'/de OR 'crossover procedure'/de OR ('randomized controlled' NEXT/1 trial*) OR rct OR 'randomly allocated' OR 'allocated randomly' OR 'random allocation' OR (allocated NEAR/2 random) OR (single NEXT/1 blind*) OR (double NEXT/1 blind*) OR ((treble OR triple) NEAR/1 blind*)	1,827,860
#8	#6 AND #7	340
#9	[adolescent]/lim OR [child]/lim OR [preschool]/lim OR [school]/lim	3,220,787
#10	#8 NOT #9	338

Search strategy in PubMed

Search	Query	Results
#1	"mesothelioma"[Title/Abstract] OR "celothelioma"[Title/Abstract] OR "cystic mesothelioma"[Title/Abstract] OR "malignant mesothelioma" [Title/Abstract] OR "mesothelial neoplasms"[Title/Abstract] OR "mesothelioma"[Title/Abstract] OR "mesotheliomatosis"[Title/Abstract] OR mesotheli* neoplasm*[Title/Abstract] OR neoplasm* mesotheli* [Title/Abstract] OR mesotheli* malignant[Title/Abstract] OR malignant mesotheli*[Title/Abstract]	18,014
#2	"Mesothelioma"[MeSH Terms]	14,759
#3	#1 OR #2	20,335
#4	"relapse*"[Title/Abstract] OR "refractory"[Title/Abstract] OR "progress*" [Title/Abstract] OR "second*"[Title/Abstract] OR "third*"[Title/Abstract]	3,506,709
#5	"Pemetrexed"[Title/Abstract] OR "alimta"[Title/Abstract] OR "Pemetrexed"[MeSH Terms]	3,798
#6	#3 AND #4 AND #5	372
#7	(randomized controlled trial[pt] OR controlled clinical trial[pt] OR randomized[tiab] OR placebo[tiab] OR clinical trials as topic[mesh:noexp] OR randomly[tiab] OR trial[ti])	1,401,059
#8	#6 AND #7	114
#9	("infant"[mesh] OR "child"[mesh] OR "adolescent"[mesh] OR adolescent[Title/Abstract] OR child*[Title/Abstract] OR infant[Title/Abstract] OR newborn[Title/Abstract])	4,193,160
#10	#8 NOT #9	113
